# Differential Effects of Angry Faces on Working Memory Updating in Younger and Older Adults

**DOI:** 10.1037/pag0000262

**Published:** 2018-06

**Authors:** Natalie Berger, Anne Richards, Eddy J. Davelaar

**Affiliations:** 1 Department of Psychological Sciences, Birkbeck, University of London

**Keywords:** working memory updating, aging, task relevance of emotion, emotional lures, *n*-back task

## Abstract

Research suggests that cognition-emotion interactions change with age. In the present study, younger and older adults completed a 2-back task, and the effects of negative stimuli were analyzed as a function of their status in the *n*-back sequence. Older adults were found to benefit more from angry than from neutral probes relative to younger adults. However, they were slower when lures were angry and less accurate when lures and probes had the same emotion. The results suggest that recollection of the *n*-back sequence was reduced in older adults, making them more susceptible to the facilitating and impairing effects of negative emotion.

Research suggests that emotion can have not only enhancing but also impairing effects on cognition (e.g., Dolcos, Iordan, & Dolcos, 2011). The dual-competition model (Pessoa, 2009, 2017) was developed to explain these contrasting effects of emotion on executive functions that are needed to manipulate content in working memory (WM; Miyake & Friedman, 2012; Miyake et al., 2000). It suggests that arousal and task relevance modulate the effect of emotion irrespective of its valence: Mildly arousing and task-relevant emotion improves executive functioning through additional recruitment of resources, whereas highly arousing and task-irrelevant emotion impairs it through the detraction of resources from the ongoing task.

However, whereas there is evidence that positive emotion can improve older adults’ WM performance (e.g., Mikels, Larkin, Reuter-Lorenz, & Carstensen, 2005), impairing effects of negative emotion were found, even if it was task relevant. For instance, when asked to bind negative and neutral pictures with their locations on the screen, lower accuracy for negative relative to neutral pictures was found in older but not younger adults (Borg, Leroy, Favre, Laurent, & Thomas-Antérion, 2011). Moreover, task-irrelevant negative material was found to impair performance in older but not in younger adults in a delayed-response WM task (Truong & Yang, 2014). Overall, it appears that negative emotion can have more detrimental effects on WM performance in aging. However, age-related changes in emotion-cognition interactions are not considered by theories such as the dual-competition model.

The socioemotional selectivity theory (SST; Carstensen, 1993) is often used to explain age-related changes in emotion-cognition interactions. It suggests that older adults allocate more cognitive resources than younger adults to emotional and specifically to positive material to enhance their well-being (for a review, see Reed & Carstensen, 2012). For instance, Borg et al. (2011) interpreted a disruption of WM binding processes for negative relative to neutral items in line with the SST as evidence for older adults’ greater focus on emotion. However, the SST posits that an emotional bias in aging requires cognitive resources (e.g., Mather & Knight, 2005) and that it can be supplanted by specific task goals (Reed & Carstensen, 2012). Given that WM tasks target a limited capacity system (Baddeley, 2003; Baddeley & Hitch, 1974) and that they are usually associated with specific instructions, it is not clear whether results from the domain of WM can be interpreted within the SST. It is also unclear how impairments through negative items can be explained by a focus on emotional and particularly positive material in aging.

Instead, cognitive changes might explain the differential effects of negative emotion on WM performance in aging. Negative material is associated with greater informational value and cognitive cost than neutral or positive material (Ito, Larsen, Smith, & Cacioppo, 1998; Labouvie-Vief, 2003, 2009; Peeters & Czapinski, 1990), which could make its manipulation in WM more difficult. Given that aging is associated with limited cognitive resources (e.g., Braver & West, 2008; Phillips & Henry, 2008) and impairments in the ability to manipulate content in WM (Reuter-Lorenz & Sylvester, 2005), greater cognitive costs of negative items are more difficult to meet in aging. Moreover, tasks with anger-inducing stimuli might be particularly challenging for older adults: Difficulties to recognize angry expressions in aging were linked to changes in frontal and temporal brain areas and in neurotransmitters (Ruffman, Henry, Livingstone, & Phillips, 2008). Older adults were also found to report less anger in response to anger-eliciting stimuli relative to younger adults, whereas no changes were found for other negative emotions such as sadness (for a review, see Kunzmann, Kappes, & Wrosch, 2014). These changes might add to age-related difficulties in cognitive tasks with angry faces.

The present study assessed the effects of angry faces on WM updating, which is known to undergo age-related changes (e.g., De Beni & Palladino, 2004; Schmiedek, Li, & Lindenberger, 2009). The *n*-back task (Kirchner, 1958) was used, in which a sequence of items is presented one at a time. For each item, participants indicate whether it is the same (on match trials) or different (on nonmatch trials) as the one presented *n* trials earlier. The task requires flexible binding and unbinding of items and contexts (Oberauer, 2009; Szmalec, Verbruggen, Vandierendonck, & Kemps, 2011), as a set of *n* most recently presented items needs to be maintained while simultaneously processing new items and updating the set. After a response to the current item (i.e., probe), the former *n*-back target becomes irrelevant, the former *n*-1 back item becomes the new *n*-back target, and items within the *n*-back buffer are maintained as future targets (McElree, 2001). Thus, the effects of material that is relevant (i.e., probe, *n*-back target) or irrelevant (i.e., *n*-1 or *n*+1 lure) for the current trial (henceforth trial relevant and trial irrelevant) can be tested. Because relevance modulates the effects of emotion on cognition (Pessoa, 2009, 2017), considering trial-relevant and trial-irrelevant emotion can contribute to a more comprehensive understanding of how emotion-cognition interactions change with age.

A previous study (Berger, Richards, & Davelaar, 2017) assessed the role of emotion on *n*-back performance in aging with a focus on probe emotion only. It was found that angry probes affected older adults’ performance differently on match and nonmatch trials. Older but not younger adults’ responses were faster for negative relative to neutral faces on nonmatch trials, whereas responses were slowest on match trials with angry faces in both groups. Because updating (i.e., replacement, overwriting) of WM content is needed on nonmatch but not on match trials (Verhaeghen & Basak, 2005), the results could indicate that negative probes facilitated updating in older adults. However, it is not clear whether updating was completed by the time a response was made, and it is possible that angry probes facilitated only subprocesses that are relevant for WM updating rather than updating in general. Indeed, auxiliary analyses showed that angry faces facilitated older adults’ performance only when they were nonmatch probes but not when they were nonmatch targets, although both were relevant for a response.

Considering age-related changes in WM updating could help to interpret this pattern of findings. Research suggests that aging is associated with reduced recollection and greater reliance on familiarity in WM updating (Schmiedek, Li, & Lindenberger, 2009). Angry faces might have signaled a nonmatch to a greater extent relative to neutral faces because of higher informational value, contributing to faster nonmatch responses rather than more efficient updating in older adults. In contrast, research has shown that older adults have difficulties to unbind task-irrelevant emotional information in an *n*-back task but only under high load (Pehlivanoglu, Jain, Ariel, & Verhaeghen, 2014), which could be due to the cognitive cost associated with negative material. Szmalec et al. (2011) suggested that inefficient (un-)binding during the WM updating process could make participants more susceptible to interference from lures and that controlled recollection processes were needed to reduce this interference. With reduced recollection in WM updating, this control process can be expected to be less efficient in older adults, making them more susceptible to interference from negative relative to neutral lures.

Thus, both facilitating and impairing effects of angry faces on WM updating can be expected because of reduced recollection in aging. However, the differential nature of negative emotion is potentially overlooked when only the effects of probes on *n*-back performance are considered. The aim of this 2-back study was therefore to analyze the effects of angry faces on WM updating in aging as a function of their status as the probe, the 2-back target or the irrelevant 1-back or 3-back lure. Because updating rather than maintenance of WM content was of interest, the design and analyses focused on nonmatch trials. The following hypotheses were tested: (1) Angry faces will facilitate nonmatch responses in terms of faster reaction times (RTs) relative to neutral faces in older but not in younger adults when they are probes, whereas no facilitating effect is expected for 2-back targets and (2) angry 1-back and 3-back lures will slow down nonmatch responses relative to neutral lures in older but not younger adults.

## Method

### Participants

Thirty-one younger (18–40 years old) and 31 older adults (60–78 years old) took part in the study. The sample size was determined on the basis of prior work (Berger et al., 2017) and allowed for a high power (.95) to detect an interaction of a small to medium effect size (.20) at α = .05 (Faul, Erdfelder, Lang, & Buchner, 2007). One older adult’s data were excluded because of accuracy at chance level (see Table 1 for details of the remaining participants). Younger adults were students at Birkbeck, University of London, and older adults were recruited from the University of the Third Age in Greater London. All reported to be in good health and to have normal or corrected-to-normal vision and hearing. Older adults scored 27 or above on the Mini-Mental State Examination (Folstein, Folstein, & McHugh, 1975). They scored higher on the National Adult Reading Test (Nelson & Willison, 1991), suggesting better vocabulary knowledge, and lower on the Digit Symbol Substitution Test from the WAIS–R (Digit Symbol; Wechsler, 1955), suggesting slower processing speed. The Ethics Board of Birkbeck approved the procedure prior to the start of the study and each participant provided written informed consent.[Table-anchor tbl1]

### Materials and Procedure

**Figure 1 fig1:**
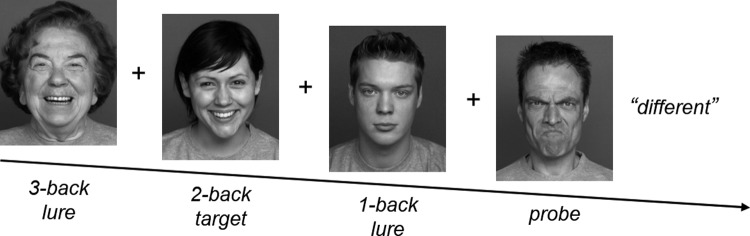
Example of a nonmatch trial sequence with angry probe and neutral 1-back lure. The remaining faces of the sequence, namely the 2-back target and the 3-back lure, are happy and constitute unscored filler trials.

Stimuli consisted of 72 faces from the FACES database (Ebner, Riediger, & Lindenberger, 2010) and were the same as those used by Berger et al. (2017). There were 14 trial sequences (see Table 2) with a balanced occurrence of neutral and angry probes, 2-back targets or 1-back and 3-back lures. Happy faces were used as unscored fillers for the sequences that were concatenated randomly. Unscored filler match trials were added to balance same and different responses. In each trial, a fixation cross was shown for 500 ms, a face for 2000 ms, and a blank screen for 200 ms. Participants compared the probe emotion (angry, neutral, happy) with the target emotion presented two trials earlier by pressing the labeled keys S for same (match trials) and D for different (nonmatch trials). The face remained on the screen for 2000 ms, even after response. The task consisted of 576 trials (see Figure 1 for an example sequence), separated into 12 blocks of 48 items. In each block, there were 14 angry, 14 neutral, and 20 happy faces. Each of the angry and neutral faces was shown on average seven times, whereas each of the happy faces was shown on average 10 times. In each block, participants started responding from the third face on, and thus, there were 46 responses per block, 23 of which were nonmatch responses.[Table-anchor tbl2][Fig-anchor fig1]

## Results

Responses and RTs were recorded for each trial. RTs faster than 200 ms or 2.5 *SD* above or below the respective group’s *M* were excluded, resulting in an exclusion^1^ of 1.4% of trials for younger and 0.70% for older adults. To assess the effects of probes and lures, mean accuracy scores and median RTs for sequences 2, 4, 5, 7, 9, 11, 12, and 14 were submitted to a four-way, mixed-factors ANOVA including the within-subject factors probe (angry, neutral), lure (angry, neutral), and position of lure (1-back, 3-back) as well as the between-subject factor of age (younger, older). To assess the effects of 2-back targets, accuracy scores and RTs were averaged across sequences 6 and 13 with angry targets and across sequences 3 and 10, with neutral targets and submitted to a two-way, mixed-factors ANOVA including the within-subject factor target (angry, neutral) and the between-subject factor of age (younger, older).

### Angry Versus Neutral Probes and Lures

#### Accuracy

**Figure 2 fig2:**
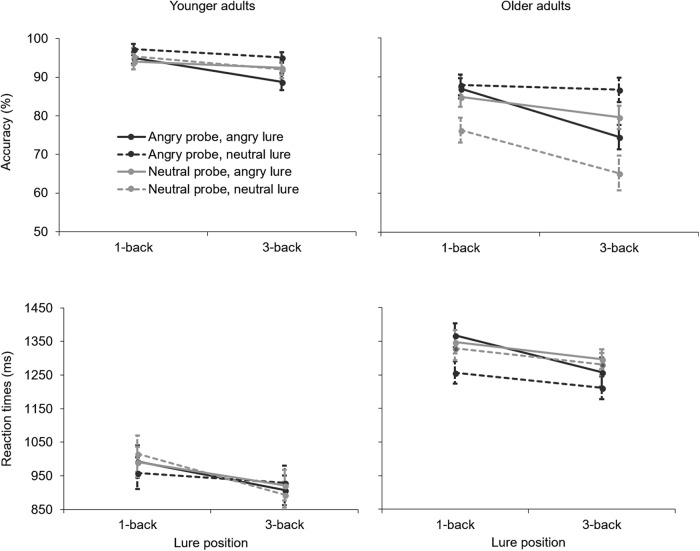
Accuracy (upper panel) and RTs (lower panel) for probes as a function of the 1-back and 3-back lures. Younger adults’ data are presented on the left and older adults’ data are presented on the right.

Accuracy for probes as a function of the 1-back or 3-back lure is shown in the upper panel of Figure 2. The four-way omnibus ANOVA revealed a Probe × Age interaction, *F*(1, 59) = 8.17, *MSE* = .01, *p* = .006, partial η^2^ = .12, which qualified a main effect of probe, *F*(1, 59) = 11.44, *MSE* = .01, *p* = .001, partial η^2^ = .16. Post hoc *t* tests showed that, consistent with hypothesis 1, older adults were more accurate when probes were angry (*M* = .84, *SD* = .11) rather than neutral (*M* = .77, *SD* = .14), *t*(29) = 4.02, *p* < .001. No such effect was found in younger adults (*p* = .683). There was no main effect of lure (*p* = .900), but there was a Lure × Age interaction, *F*(1, 59) = 8.44, *MSE* = .01, *p* = .005, partial η^2^ = .13, and a Probe × Lure interaction, *F*(1, 59) = 19.70, *p* < .001, partial η^2^ = .25. These were qualified by a Probe × Lure × Age interaction, *F*(1, 59) = 7.51, *p* = .008, partial η^2^ = .12. Separate analyses for the two age groups revealed a Probe × Lure interaction in older adults, *F*(1, 29) = 17.82, *p* < .001, partial η^2^ = .38, but not in younger adults (*p* = .126). Post hoc *t* tests showed that older adults were less accurate when probe and lure had the same emotion: For neutral probes, they were less accurate when the lure was also neutral (*M* = .72, *SD* = .17) rather than angry (*M* = .82, *SD* = .13), *t*(29) = 4.22, *p* < .001. Similarly, for angry probes, they were less accurate when the lure was also angry (*M* = .81, *SD* = .10) rather than neutral (*M* = .87, *SD* = .15), *t*(29) = 2.74, *p* = .010.[Fig-anchor fig2]

There was also a Probe × Lure × Position of lure interaction, *F*(1, 59) = 7.51, *p* = .008, partial η^2^ = .12, which qualified the Probe × Lure interaction. Separate analyses for trials with 1-back and 3-back lures revealed a Probe × Lure interaction for 3-back lures, *F*(1, 60) = 20.83, *p* < .001, partial η^2^ = .26, but not for 1-back lures (*p* = .090). Post hoc *t* tests showed that for angry probes, accuracy was lower when the 1-back lure was also angry (*M* = .82, *SD* = .17) rather than neutral (*M* = .91, *SD* = .14), *t*(60) = 3.98, *p* < .001. For neutral probes, accuracy was lower when the 3-back lure was also neutral (*M* = .80, *SD* = .22) rather than angry (*M* = .86, *SD* = .15), *t*(60) = 3.19, *p* = .002, highlighting that a 3-back lure of the same emotion as the probe affected responses. There was also a main effect of age, *F*(1, 59) = 28.81, *p* < .001, partial η^2^ = .33, with lower accuracy in older (*M* = .81, *SD* = .12) than in younger adults (*M* = .94, *SD* = .07). No further effects were observed.

#### Reaction times

RTs for probes as a function of the 1-back or 3-back lure are presented in the lower panel of Figure 2. The four-way omnibus ANOVA revealed a main effect of probe, *F*(1, 59) = 4.77, *MSE* = 19,160, *p* = .033, partial η^2^ = .08, with faster RTs for angry (*M* = 1110 ms, *SD* = 268 ms) than for neutral probes (*M* = 1135 ms, *SD* = 267 ms). Consistent with hypothesis 2 predicting impairing effects of angry lures in older adults, there was a Lure × Age interaction, *F*(1, 59) = 5.73, *p* = .020, partial η^2^ = .09, qualifying the main effect of lure, *F*(1, 59) = 7.71, *MSE* = 11,530, *p* = .007, partial η^2^ = .12. Post hoc *t* tests revealed that older but not younger adults (*p* = .766) were slower when lures were angry (*M* = 1318 ms, *SD* = 160 ms) rather than neutral (*M* = 1271 ms, *SD* = 156 ms), *t*(29) = 3.15, *p* = .004. There was a main effect of lure position, *F*(1, 59) = 31.76, *MSE* = 17,639, *p* < .001, with slower RTs for 1-back (*M* = 1157 ms, *SD* = 267 ms) than for 3-back lures (*M* = 1087 ms, *SD* = 267 ms). There was also a main effect of age, *F*(1, 59) = 43.45, *MSE* = 323077, *p* < .001, with slower RTs in older (*M* = 1294 ms, *SD* = 152 ms) than in younger adults (*M* = 951 ms, *SD* = 238 ms). No further effects were observed.

### Angry Versus Neutral Targets

#### Accuracy

The two-way ANOVA revealed a main effect of target, *F*(1, 59) = 19.75, *MSE* = .01, *p* < .001, partial η^2^ = .25, with lower accuracy for angry (*M* = .83, *SD* = .14) than for neutral targets (*M* = .89, *SD* = .12 *t*(60) = 4.43, *p* < .001. There was also a main effect of age, *F*(1, 59) = 11.10, *MSE* = .05, *p* = .001, with lower accuracy in older (*M* = .81, *SD* = .13) than in younger adults (*M* = .91, *SD* = .08). No further effects were observed.

#### Reaction times

The two-way ANOVA revealed a main effect of target, *F*(1, 59) = 14.80, *MSE* = 8894, *p* < .001, partial η^2^ = .20, with slower RTs for angry (*M* = 1140 ms, *SD* = 259 ms) than for neutral targets (*M* = 1083 ms, *SD* = 230 ms). There was also a main effect of age, *F*(1, 59) = 50.50, *MSE* = 124298, *p* < .001, with slower RTs in older (*M* = 1274 ms, *SD* = 135 ms) than younger adults (*M* = 953 ms, *SD* = 208 ms). No further effects were observed.

## Discussion

The aim of this study was to assess the effects of negative emotion on WM updating in aging. In a 2-back task, angry faces were the probe, the 2-back target or the 1-back or 3-back lure. Replicating previous findings, older adults were found to benefit from angry relative to neutral probes when detecting a nonmatch: Although both age groups were faster when probes were angry rather than neutral, only older adults were also more accurate. In contrast, no facilitating effect was found for angry 2-back targets as both age groups were less accurate and slower on nonmatch trials with angry relative to neutral targets. Moreover, older but not younger adults were slower when lures were angry rather than neutral, and they were less accurate when lures and probes had the same emotion. Lastly, no-longer-relevant lures affected WM updating because accuracy decreased when 3-back lures and probes had the same emotion. Overall, the results are in line with previous work showing older adults’ increased reliance on familiarity in WM updating (Schmiedek, Li, & Lindenberger, 2009). The present results extend previous research by showing that this makes older adults susceptible to the facilitating and impairing effects of negative emotion.

The present study is the first to show that angry faces can both improve and interfere with older adults’ *n*-back performance, and that the effect depends on the item’s status as probe or lure. Although all participants but particularly older adults benefited from angry probes on nonmatch trials, the benefit did not extend to angry 2-back targets that were also trial relevant. This suggests that angry faces did not improve WM updating in general. Whereas updating (i.e., replacement, overwriting) is required on nonmatch trials (Verhaeghen & Basak, 2005), it was not completed by the time of response as 3-back lures affected responses. Instead, it appears that angry probes facilitated processes specific to nonmatch responses in aging. Schmiedek, Li, and Lindenberger (2009) argued that older adults relied on familiarity in WM updating because of reduced recollection. It is possible that angry faces were more distinct and signaled a nonmatch to a stronger degree than neutral faces, thus facilitating older adults’ responses to angry probes. This is in line with findings that angry faces are easily detected among distractors (e.g., Hansen & Hansen, 1988). In contrast, it appears that younger adults, who showed high accuracy, did not rely on emotional cues because of effective recollection of the *n*-back sequence. Although near-ceiling accuracy might have masked better performance for angry probes in younger adults, the finding of greater benefits from angry probes in older adults is in line with previous research (Berger et al., 2017).

Moreover, the results showed that angry faces interfered with older adults’ WM updating when they were lures as they slowed down responses. Older adults were also less accurate when presented with lures that had the same emotion as the probes. Again, these results are compatible with the notion that older adults rely on familiarity in WM updating, as research suggests that controlled recollection processes were needed to reduce interference from lures in WM updating (Szmalec et al., 2011). It appears that a clear assignment of the items to their serial position and a distinction between task-relevant and task-irrelevant material was unavailable to older adults. However, the results of the present study extend previous research by showing that age-related difficulties in WM updating are exacerbated by negative emotion, making older adults susceptible to interference from angry lures.

It is likely that greater interference from angry lures in older adults’ WM updating was due to the cognitive cost of negative and particularly threatening material. According to the dual-competition model (Pessoa, 2009, 2017), highly arousing threat can detract cognitive resources that are needed for WM operations. However, the present research suggests that even mildly threatening items such as pictures of angry faces can interfere with WM updating in older adults. It is possible that they were less able to compensate for the detraction of resources in the presence of angry faces than younger adults because of limited cognitive resources (e.g., Braver & West, 2008; Phillips & Henry, 2008), which resulted in a slowdown in performance. The findings are in line with research showing greater disruptive effects of negative items on WM in older relative to younger adults (Borg et al., 2011; Truong & Yang, 2014). Moreover, the findings highlight the need to adapt theories on the interplay between emotion and executive control such as the dual-competition model (Pessoa, 2009, 2017) to account for age-related changes.

Previous studies (e.g., Borg et al., 2011) have interpreted age-related changes in emotion-cognition interactions in WM as evidence for older adults’ focus on emotion in line with the SST (Carstensen, 1993). The SST suggests that older adults focus on positive material to enhance their well-being when sufficient resources are available, and their motivational goals are not supplanted by task goals. In the present study, however, age-related changes emerged for angry faces in a resource-demanding WM task. Given that WM is a limited capacity system (Baddeley, 2003; Baddeley & Hitch, 1974) and that specific task instructions were used, it can be assumed that the scope to process items freely was limited. Thus, a motivation-based approach seems unsuitable to explain the pattern of findings observed in this study given that some core assumptions of the SST were not met in the present research.

Finally, lower accuracy in both age groups was observed when the probe and the 3-back lure had the same emotion, suggesting that outdated items were not discarded from WM. Interference from 3-back lures in a 2-back task could be due to rehearsal as suggested by Szmalec et al. (2011), who found stronger interference from older relative to more recent lures in a 3-back task. The authors suggested that interference was stronger from older lures because they had been rehearsed more often over the course of the sequence. Overall, the finding suggests that WM updating is a complex process that is not completed at the time of response in an *n*-back task. It also highlights that a comprehensive analysis including probes, lures, and targets is needed to understand how subprocesses involved in WM updating interact with emotion.

In summary, the present study contributed to research differentiating between enhancing and impairing effects of emotion on WM updating and showed that older adults are more susceptible to these effects than younger adults. The findings highlight the need to assess the effects of emotional probes, targets, and lures to understand how emotion affects WM updating. Moreover, the research suggests that existing theories on the interplay between emotion and executive functions need to be adapted to account for age-related changes.

## Figures and Tables

**Table 1 tbl1:** Participant Characteristics

Variable	Younger adults		Older adults		Group difference	
	*M*	*SD*	*M*	*SD*	*t*	*p*
Age, years	25.03	5.57	68.60	4.43		
Gender, male/female	11/20		10/20			
Education, years	16.73	2.45	16.50	3.16	.31	.756
NART verbal IQ	106.82	8.05	118.80	4.07	−7.08	<.001
Digit symbol	73.84	8.76	52.53	9.28	9.22	<.001
BDI II	4.65	4.92	4.23	3.54	.77	.446
STAI trait anxiety	34.13	7.44	33.49	7.14	.39	.698
MMSE			29.38	.73		
*Note.* NART = National Adult Reading Test; BDI II = Beck Depression Inventory II; STAI = State-Trait Anxiety Inventory; MMSE = Mini-Mental State Examination.						

**Table 2 tbl2:** Trial Sequences

No	Lure 1-back	Target 2-back	Lure 1-back	Probe
1	Happy	Happy	Happy	**Neutral**
2	Happy	Happy	**Neutral**	**Neutral**
3	Happy	**Neutral**	Happy	**Neutral**
4	**Neutral**	Happy	Happy	**Neutral**
5	Happy	Happy	**Angry**	**Neutral**
6	Happy	**Angry**	Happy	**Neutral**
7	**Angry**	Happy	Happy	**Neutral**
8	Happy	Happy	Happy	**Angry**
9	Happy	Happy	**Neutral**	**Angry**
10	Happy	**Neutral**	Happy	**Angry**
11	**Neutral**	Happy	Happy	**Angry**
12	Happy	Happy	**Angry**	**Angry**
13	Happy	**Angry**	Happy	**Angry**
14	**Angry**	Happy	Happy	**Angry**
*Note.* Bold font indicates critical angry and neutral trials among happy filler trials.				

## Footnotes

1 To check the robustness of findings obtained with this method of excluding improbable RTs, all analyses were repeated with an alternative method of excluding participants on the basis of group means. The results remained qualitatively the same and significant.
